# Mr. Jones's Additional Test for Arsenic

**Published:** 1810-10

**Authors:** Thomas Jones

**Affiliations:** Howden


### *To the Editors of the Medical and Physical Journal.*

GENTLEMEN,

IN compliance with the wish of your correspondent, Mr. Hume, I have made the following additional experiments with the powder found in the stomach of the young woman who died by poison. These experiments with those made before, are, in my opinion, decisive proofs that the powder in question does not contain either arsenic or corrosive sublimate: they moreover tend to establish the opinion that though a person may die from the effects of poison; yet in consequence of vomiting, purging, and taking diluents, it is not always possible to detect it in the stomach after death.

It was with the view of confirming this opinion, that I was prevailed upon to trouble you with the report; two grains of the powder, with eight ounces of rain water, were boiled in a Florence flask, which was shook frequently, both before and after the addition of one grain of subcarbonate of potash. The powder however still appeared undissolved at the bottom of the vessel.

Lunar Caustic was applied, as directed by Mr. Hume, to to the surface of a part of this fluid. A *white* cloud was instantly perceived: a second touch produced a white precipitate, which turned grey on standing till the following morning.

A similar experiment made with arsenic produced a yellow precipitate.

To another portion of the fluid the sulphat of copper in substance was applied: this produced a light blue semitransparent liquor which deposited slowly a blue cloud. A similar experiment made with arsenic produced a semitransparent liquor of a pea green. On the addition of lime water to another portion of the fluid it became rather milky. The remaining part of the fluid was filtered: the powder was collected and dried on paper. It weighed exactly two grains, not having lost any of its weight by boiling; and is, without doubt, sand with which the poisonous substance was mixed.

I afterwards made the two experiments recommended by your correspondent in the hundred and thirty-eighth number of your Journal: to which, in order to avoid being prolix, I refer your readers for the detail.

In the first experiment, which was made exactly as directed by Mr. Hume, no nitrous fumes were evolved; a slight *white* smoke only being perceived. In the second, on the application of the caustic no perceptible change was produced.

I am, &c. THOMAS JONES.

